# A reflection on salts dissolved in salts: ionic liquid mixtures – ion pairs, ion pairing and ionic liquids

**DOI:** 10.1039/d5sc90213d

**Published:** 2025-10-06

**Authors:** Tom Welton

**Affiliations:** a Department of Chemistry, Imperial College London London UK t.welton@imperial.ac.uk

## Abstract

This commentary, which builds upon the original *Chemical Science* article (M. Y. Lui, L. Crowhurst, J. P. Hallett, P. A. Hunt, H. Niedermeyer and T. Welton, *Chem. Sci.*, 2011, **2**, 1491–1496, https://doi.org/10.1039/c1sc00227a) published in 2011, discusses the evidence for the formation (or not) of ion pairs and the development of the concept of ion pairing in ionic liquids.

## Abstract 2

In celebration of our 15th anniversary and some of our most popular articles, Tom Welton reflects on ionic liquid mixtures, following on from the original study by J. P. Hallett and T. Welton *et al.* (*Chem Sci.*, 2011,https://doi.org/10.1039/C1SC00227A).

## Graphical abstract

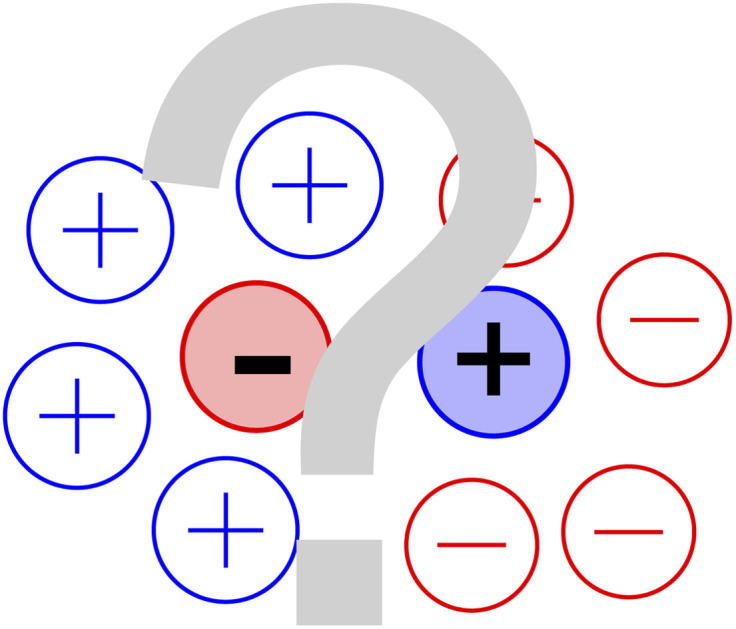

Fifteen years ago, colleagues and I published the article *Salts dissolved in salts: ionic liquid mixtures* (https://doi.org/10.1039/c1sc00227a),^1^ in which we studied the spectra of the empirical solubility probe 1-ethyl-4-(methoxycarbonyl)pyridinium iodide (Kosower's salt, [Py]I) in a number of ionic liquids (ILs). This salt had been used historically to investigate the polarity of molecular solvents by measuring the wavelength of its UV-vis absorption maximum.^2^ This absorption arises from an anion to cation charge transfer, which can only occur when these ions are in direct contact. Hence, this provided a means to study ion pairing in ILs. By measuring the changing extinction coefficient of this absorption as a function of concentration of the salt, we could determine the thermodynamics of the interactions of these solute ions in the ILs. We found that although such contacts did occur, the Gibbs free energy of the metathesis reaction taking place in the IL solution (eqn (1), where [cat]^+^ and [an]^−^ are the cation and anion of the IL, respectively) is essentially zero.

1 [Py]I + [cat][an] ⇌ [Py][an] + [cat]I** ***K*_eqm_ ≈ 1,** **Δ*G*_eqm_ ≈ 0

We concluded that the lack of energetic consequence of this change meant that the random, statistical contact of the solute ions in ILs could not be described as ion pairing. The invitation to return to and reflect on this paper led me to consider what has happened regarding the question of whether ion pairs (IPs) form in ILs in the intervening years. This commentary is not intended to be a comprehensive review of the area, which would require much greater length than this format allows. I have attempted to select some representative papers, and I apologise if I have not cited your work; this is in no way to be taken as an indication of quality. More thorough reviews are available for those who wish to know more on this interesting subject.^3–5^

## Early indications of ion pairing in ionic liquids

The origin of thinking about the presence of IPs in ILs arose from their surprisingly low conductivities. One would expect a liquid that is composed entirely of ions to be highly conducting, but ILs are often less conducting than ionic solutions in molecular solvents. Most ILs are highly viscous;^6^ perhaps this could explain their poor conductivity. However, the Walden plots (log equivalent conductivity *vs.* log reciprocal viscosity) of ILs show that even when viscosity has been taken into account ILs have lower conductivity than expected.^7^ Further to this, the concept of *ionicity* was introduced to describe the lower conductivity of ILs than expected from the diffusion coefficients of their constituent ions.^8^ The simplest explanation for this was that the ILs contained a significant number of IPs, the movement of which did not contribute to charge transport. There are other possible explanations for this, which I will not go into here, such as charge transfer between ions leading to these having charges of less than one.^9^

Using surface force apparatus,^10^ the Israelachvili group showed that forces between the surfaces had an extraordinary long decay length, which is usually associated with very dilute electrolytes. It was suggested that this arose through the formation of IPs in the ILs, with there being very few free ions. This was, to say the least, a very controversial claim,^11^ although the idea has been supported computationally using a simplified model of an IL, which concluded that it was composed primarily of dipolar IPs with relatively few free ions.^12^ However in a subsequent paper, they clearly noted that the ions in the IL do not form classic IPs in which each ion exclusively pairs with only one specific counterion but rather suggested that local neutrality is driven by strong coulombic interactions between the IL's ions.^13^

None of these studies involved direct observations of IPs, but instead were measurements of bulk properties that were interpreted by reference to a concept that had been developed to understand behaviours of dilute solutions of electrolytes in molecular solvents—ion pairing.

## The ion pair concept

**Fig. 1:**
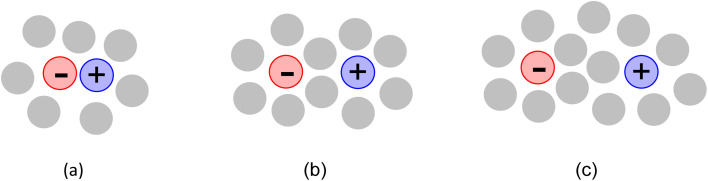
(a) Contact IP (b) solvent-shared IP and (c) solvent-separated IP in molecular solvents.

In molecular solvents, the concept of ion pairs is well-established.^14^ They can range from contact IPs in which the ions are in direct contact, to solvent-shared IPs in which the two ions are separated by molecules of solvent that are in the first solvation sphere of both ions, and then solvent-separated IPs in which both ions are fully solvated but remain in otherwise close contact (Fig. 1). These kinds of IPs will occur, to differing extents depending upon the polarity of the solvent and the relative strength of ion–ion and ion–solvent interactions, when ILs are dissolved in molecular solvents, but these solutions are not the subject being discussed here. Nor are other systems in which molecular species are present, such as deep eutectic solvents,^15^ solvate ILs,^16^ or protic ILs for which there is evidence of incomplete ionisation.^17^

We need to think about how this concept transfers into ILs and what we mean when we say ‘ion pair’ in relation to ILs. These should be composed of ions and they should be a pair, that is *two*. Higher order clusters of ions are not IPs. It follows that, evidence of ion association is not necessarily evidence for ion pairing.

This second criterion immediately causes a problem for ILs, in which all the ions are surrounded by other ions. In addition, it had already been demonstrated that ion clusters, such as [(cat)_*q*+1_(an)_*q*_]^+^ and [(cat)_*q*_(an)_*q*+1_]^−^, could be observed in the mass spectra of ILs.^18^ The presence of such clusters could explain both the Walden plots and the ionicity results. However, mass spectrometry is blind to the presence of neutral species, so this is not strong evidence for the absence of IPs. Also, these clusters were detected in the high vacuum of the spectrometer and not directly in the ILs, in which they might not be present as distinct species. The presence of such clusters has also been invoked to explain the fluorescence of the organic probe 2-amino-7-nitrofluorene in 1-butyl-3-methylimidazolium hexafluorophosphate ([C_4_C_1_im][PF_6_]), but this was indirect evidence for these.^19^

To describe the interaction between two particular ions in an IL as an IP there needs to be some way in which this partnership is different, even preferential or in some way special, when compared to other interionic interactions in the system. If one accepts this need for difference, then it could be argued that there can be no IPs in a neat simple (composed of just one type of cation and one type of anion) IL. If all the cations or anions are the same and interchangeable, then all the interionic interactions must ultimately be the same. However, this has not been how the discussion in the literature has developed. Due to the difficulty in obtaining direct observations of specific IPs in neat simple ILs, research in this area has been dominated by computational investigations.

A very common claim for the presence of IPs in ILs comes from using *ab initio* quantum mechanical density functional theory (DFT) calculations to interpret experimentally observed phenomena. If the observed behaviour can be sufficiently explained from the results of DFT calculations that show strong interactions between [cat]^+^ and [an]^−^, then the behaviour is often attributed to the formation of IPs. However, this is highly problematic. The computational cost of DFT calculations is such that it is necessary to restrict the size of the studied system, consequently most of these studies include just one cation and one anion in the calculations.^20^ This forces the formation of an IP. However, studies in which many possible configurations of these are explored usually find that there are several stable structures that are sufficiently low in energy that they will all likely be present in the IL. While studies such as this can provide a great deal of insight into the behaviours of ILs, they can say nothing about the presence of IPs in the bulk IL.

It is possible to apply *ab initio* calculations to larger clusters of ions and when this has been done, there has been no evidence for a specific IP to be formed within the cluster.^21,22^ For example, Ekaterina Izgorodina and Su Chen used calculations on clusters from 1 cation and 1 anion up to 16 cations and 16 anions to predict ^1^H NMR chemical shifts of ILs with 1-ethyl-3-methylimidazolium cation ([C_2_C_1_im]^+^) and various anions.^23^ Not only was there no evidence for discrete IPs within the clusters, they found that larger clusters were required to achieve accurate predictions, with the poorest results coming from the calculations for a single pair of ions.

## Is it just a matter of timing?

Some way of defining an IP in an IL is required. Our work was based upon a thermodynamic description of IPs.^1^ I would argue that this is the strongest criterion that there can be. However, there are other ways in which IPs have been described, particularly temporal criteria. Two ions might be an IP if the contact could be considered in some way ‘long-lived’. This could be defined in terms of some absolute timescale (is the contact time longer than X?) or in a relative way (is the contact time longer than some other process in the system?). To use an absolute timescale would be problematic in two ways: (i) the selection of the timescale would be somewhat arbitrary and; (ii) if you picked a sufficiently short timescale almost any interaction between ions could look like ion pairing. Also, we are trying to identify an interaction of two ions that is different to all the other ion–ion interactions that are occurring in the IL. This lends itself to defining the timescale in a relative way.

Our paper also pointed to the importance of considering timescales.^1^ We noted that presence of the UV-vis absorption meant that the contact between the [Py]^+^ and I^−^ ions lasted long enough for the electronic transition to occur (*n*.*b*., this gives the minimum time that the ions could be in contact, not the actual time that they are), whereas the concentration dependence of this absorption, which results from much slower processes of ion translation, showed that *K*_eqm_ ≈ 1 for the metathesis reaction in eqn (1), so constant exchange of ions must be occurring. Hence, we reasoned that the fleeting contacts necessary to allow the charge transfer to occur could not be considered an IP. So, what would be a reasonable timescale to use?

**Fig. 2:**
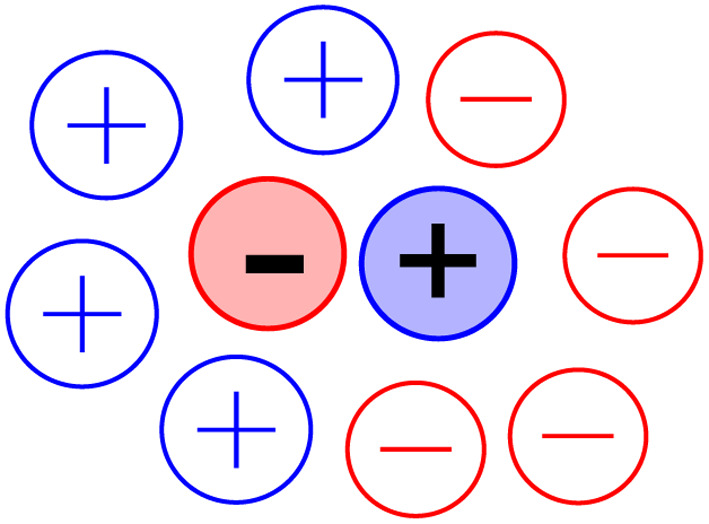
Closest contact ion pair in an IL.

First, we need to consider what is the species to which we are going to give a lifetime. This was easy for our study, the species was a solute cation ([py^+^]) in direct contact with a solute anion (I^−^), which were at sufficiently low concentration that larger clusters of these were unlikely to contribute to any spectroscopic observations. This is a more difficult question for neat simple ILs. Most researchers have used some variant of oppositely charged ions that are in the closest contact (Fig. 2). The analogy of this to contact IPs in molecular solvents is clear.^14^ Details vary, such as whether the distance is between centres of charge or mass or the closest contact atoms of each ion, but the principle remains that the IP consists of an ion and its closest counterion; when that particular counterion is no longer the closest, the IP has been broken and a new one formed.^3,24^

While for a neat simple IL there is a range of cation–anion contact times, it is not clear which of these could be described as long-lived for this purpose (*e.g.*, is greater than the mean sufficient, or some number of standard deviations above this?), and the selection would be somewhat arbitrary. For an IP to affect the conductivity of an IL it must persist long enough for ion translation to occur. Based upon molecular dynamics (MD) simulations of 1-butyl-1-methylpyrrolidinium bis(trifluoromethanesulfonyl)imide ([C_4_C_1_pyrr][NTf_2_]), Claudio Margulis and coworkers introduced the ‘cage-jump’ model of ion diffusion in ILs.^25^ In this model, each ion is confined in a ‘cage’ which is composed of oppositely charged ions and after some time it ‘jumps’ along a trajectory to enter another cage where it becomes trapped again. While trapped in the cage the ion is not contributing to diffusion and, more importantly, it is interacting with all the ions of the cage as it rapidly rattles about within it. Hence, while this is undoubtedly ion association, it cannot be described as ion pairing. It is only when it escapes the cage by this ‘jump’ that it diffuses. While, of course, the times that individual ions are resident in particular cages vary, it was shown that this was roughly of the order of 100 ps. This would seem to be a reasonable lifetime above which to define an ion–ion contact to be not just random, and so worthy of the description of being an IP in an IL.

Barbara Kirchner and coworkers have thought very deeply on this.^3,26^ They have developed the concept of comparing the lifetime of a cation–anion hydrogen bond (‘hydrogen bond lifetime’), the lifetime of a cation–anion contact (‘IP lifetime’) and the time for an ion to separate from all the ions making up its cage (‘ion cage lifetime’). Care is required when comparing these timescales, because all differ with the precise technique being used for the calculations, the IL being studied and are sensitive to temperature.^27^ Therefore, it is important to be sure that you are comparing like with like when looking at the results from different studies. Nevertheless, the ion cage lifetimes are reliably at least an order of magnitude greater (typically hundreds of ps) than the IP lifetimes for a given IL, which in turn are generally an order of magnitude greater than the hydrogen bond dynamics.^28^ Importantly, these IP lifetimes are so short that the ions generally do not have sufficient time to diffuse together.^29^ These transitory couplings would not meet the criterion of being associated long enough to ‘jump’ together.

Patricia Hunt and coworkers have studied hydrogen bonding in ILs in great detail.^30^ Hydrogen bonding in [C_*n*_C_1_im]Cl (*n* = 2 or 4), has been well studied both experimentally^31^ and theoretically. The Cl^−^ anion is a strong hydrogen bond acceptor and DFT calculations showed that it could be located in several positions around the ring, with a strong preference for forming a hydrogen bond at the C2–H position of the imidazolium ring.^32^ These calculations also demonstrated a significant coulombic contribution to the hydrogen bonds. MD simulations showed that this justified that a wider angle should be used to characterise the hydrogen bonds in ILs, and when this was done each cation hydrogen bonds to several Cl^−^ (mostly at different positions around the imidazolium ring, but in a small percentage of cases one hydrogen atom participates in two H-bonds) and each Cl^−^ anion forms multiple H-bonds to hydrogens on different cations. Thus, these hydrogen bonds are not driving the formation of an exclusive IP. Further to this, the lifetimes of the hydrogen bonds were investigated and two timescales for cation–anion hydrogen bonds were found:^33^ a shorter ‘continuous’ hydrogen bond (the time until the two atoms no longer meet the criteria for a hydrogen bond) and a longer ‘intermittent’ hydrogen bond (allowing the two ions to break and reform the hydrogen bond until they finally separate permanently). The continuous lifetime for the strongest H-bonding, C2–H⋯Cl^−^ of [C_2_C_1_im]Cl at 450 K was 6.9 ps, while the intermittent residence lifetime was 137.8 ps, over 20 times longer. When less hydrogen bond accepting anions are considered, other positions around the cation become more highly populated including the non-hydrogen bond sites above and below the plane of the ring,^34^ and hydrogen bond lifetimes become significantly shorter.^35^

Experimental studies have been performed, with mixed results. Pulsed-field-gradient NMR spectroscopy has been used to study the diffusion of ions in [C_2_C_1_im][NTf_2_] and [C_2_C_1_im][OAc] (where [OAc]^−^ is the acetate ion).^36^ It was reasoned that the different values of self-diffusion coefficients of the [C_2_C_1_im]^+^ and [NTf_2_]^−^ in [C_2_C_1_im][NTf_2_] meant that these cannot be diffusing together, whereas the very similar values for [C_2_C_1_im]^+^ and [OAc]^−^ in [C_2_C_1_im][OAc] strongly suggests that these are diffusing together. This was explained as being due to ion pairing between the [C_2_C_1_im]^+^ and [OAc]^−^ ions, driven by the strong hydrogen bonding between these. Two-dimensional infrared (2D-IR) has been used to investigate the possibility of ion pairing in the ILs [C_2_C_1_im][OAc], [C_3_C_1_im][NTf_2_], and [C_2_C_1_im][SCN].^37^ The authors argued that, since the ions are not spherical, ion pairing would lead to an anisotropic rotational ordering of neighbouring ions. Contrary to this, they found zero anisotropy in all pairs of interionic vibrational modes investigated and concluded that there was no evidence for the formation of IPs, or indeed ion clusters, in these ILs. So, one study was used to propose ion pairing in [C_2_C_1_im][OAc] and the other to refute it.

There have been examples of the use of different temporal definitions of IPs in ILs. As part of a study of high temperature molten salt systems, it was suggested that a lower limit should be imposed on the IP lifetime to avoid counting encounters that are just collisions in a dynamic system.^38^ If this were applied more generally to ILs, the removal of very brief contacts from calculations of the mean IP lifetimes would lead to these increasing. In a study of [C_4_C_1_im][MeOSO_3_] (where [MeOSO_3_]^−^ = methyl sulfate),^39^ two different definitions of IPs were used. The shorter lifetime definition (*ca*. 2 ns) is that the [C_4_C_1_im]^+^ and [MeOSO_3_]^−^ were considered to be an IP if their centres of mass were closer than 0.75 nm and the IP was broken when this was no longer the case. Using this definition the IPs broke up before they could diffuse together. The longer lifetime definition (*ca*. 15 ns) allowed the ions to move farther away from each other, so long as they return, and the IP is only considered to be broken when the separation is permanent, giving lifetimes an order of magnitude longer and sufficient for the IP to contribute to diffusion. Neither definition considers what is happening regarding other ions in the vicinity. Recently, the concept of Weak IPs (WIPs) in [C_4_C_1_im][PF_6_] has been proposed.^40^ These WIPs are not defined by closest contact, but rather that the IP is the coupling of a central ion and the counterion that continuously stays in its first solvation sphere (*i.e.*, cage) for the longest time. Ions that are momentarily in closer contact to the central ion are not considered to be IPs. This contrasts to the situation in molecular solvents, in which the IP is always the closest contact of ions of opposite charge.^14^ Using this definition, the authors calculated the average lifetime of these WIPs to be greater than 11.0 ns at 300 K, which is sufficient to allow time for diffusion to occur.

One condition in which is it accepted that IL ions do form IPs is in the gas phase when they evaporate.^41^ This begs the question of whether the IP forms in the IL and then evaporates, or only forms in the gas phase. Evaporation of ions occurs from the surface layer of the IL,^42^ the structures of which are well understood and often have an excess of one of the ions.^43^ Since the ions are in direct contact with the vacuum, approximately half of their solvation spheres/ion cages are missing. The ions are mobile and move across the surface, as well as being reabsorbed into the bulk and replaced by other ions. At some point two oppositely charged ions meet and interact sufficiently strongly to form an IP, which evaporates. Interestingly, the ILs studied,^42^ [C_2_C_1_im][NTf_2_] and [C_2_C_1_im][EtOSO_3_] (where [EtOSO_3_]^−^ = ethyl sulfate), showed no propensity for the formation of IPs in the bulk. Hence, this could be described as ion pairing *on* the ILs, rather than *in* these.

So far, I have only addressed interactions of oppositely charged ions. However, despite the strong coulombic repulsion generated by two like-charged ions, close contacts of these have been observed. Ralf Ludwig and coworkers have shown hydrogen bonding between the –OH groups of [(HO)C_2_C_1_im][BF_4_] or [(HO)C_2_C_1_im][PF_6_] (where [(HO)C_2_C_1_im]^+^ = 1-(2-hydroxyethyl)-3-methylimidazolium).^44^ Interestingly, these persisted when these ionic liquids were mixed with their alkyl counterparts [C_3_C_1_im][BF_4_] and [C_3_C_1_im][PF_6_].^45^ Anion–anion attraction has been shown for tetracyanoborate anions in ILs of these.^46^ These interactions were shown to be driven by induction and dispersion forces between the multiple cyano groups. However, even without the presence of functional groups that can drive like-charged ion attraction, evidence has been found for these. In MD simulations of [C_2_C_1_im][N(CN)_2_] and [C_2_C_1_im][OTf] it was found that the first solvation sphere of the cations contained both cations and anions and there was no evidence for preference of a cation for a single anion.^47^ Using a statistical analysis of ion association, two distinct local ionic environments, “aggregated” and “non-aggregated”, were found in a model IL, which were associated with like-charge correlations rather than unlike-charge attraction.^48^ Also it is well accepted that nanosegregation in ILs arises when the alkyl chains of the IL ions are sufficiently long for these to interact with each other, which is another example of like-charged ions interacting with each other.^49^ None of these like-charged ion interactions can be considered to be IPs.

## Solutions of salts and ionic liquid mixtures

While the concept of ion pairing in neat simple ILs requires that somehow two ions that are identical to the other ions in the liquid need to be differentiated from all the others, for solute ions this is straight-forward, since the solute ions are different to the solvent ions.

Even prior to our paper, Ruth Lynden-Bell used simulation of the potential of mean force between pairs of solute ions to show that the screening of two opposite charges is large enough in the IL [C_1_C_1_im]Cl that any solute ions that are separated by solvent ions are fully screened from each other.^50^ She further showed that it is not even necessary to have solvent ions between two solute ions for them to be highly screened. From this, we can ignore solvent-shared and solvent-separated IPs in ILs and conclude that even the closest contact IPs are generally energetically unfavoured.

With a few exceptions,^51^ ILs mix well with each other and in many cases form almost ideal mixtures.^52^ The study of these has led to hundreds of papers, too many to go into here. Structural features, such as nanosegregation and charge ordering have been seen.^53^ Preferential interactions have often been identified for good hydrogen bond donor cations (or sites on cations, such as the C2–H position of imidazolium rings) with good hydrogen bond acceptor anions, but these preferences are slight and have not been attributed to the formation of IPs, but rather an enhancement of the solvation sphere (*i.e.*, ion cage) with one of the counter ions,^54^ or even to hindered rotation within an ion cage that is composed of a statistical mix of counterions.^55^ A combined IR and NMR study of [C_8_C_1_im]_*x*_[C_4_C_1_im]_(1−*x*)_[BF_4_], [C_4_C_1_im]Cl_*x*_[BF_4_]_(1−*x*)_, and [C_4_C_1_im]Cl_*x*_I_(1−*x*)_ mixtures showed some very interesting results.^56^ The longer timescale NMR results showed the same nonlinear mixing behaviour reported in other papers, but the shorter timescale IR results demonstrated that the hydrogen bonding between the C2–H and the anions in the mixtures was simply proportional to the anion concentrations in the mixture. In a similar combined IR and NMR study of [C_2_C_1_im][NTf_2_]_*x*_[DCA]_(1−*x*)_ (where [DCA]^−^ = dicyanamide) mixtures, a number of clusters were identified.^57^ DFT calculations of clusters of up to 8 ions for mixtures of the guanidinium-based ILs [TMG(C_2_)][EtOSO_3_] (where [TMG(C_2_)]^+^ = 2-ethyl-1,1,3,3-tetramethylguanidinium) with [TMG(C_2_)][NTf_2_], and [TMG(C_2_)_2_][EtOSO_3_] (where [TMG(C_2_)_2_]^+^ = 2,2-diethyl-1,1,3,3-tetramethylguanidinium) with [TMG(C_2_)_2_][NTf_2_] have shown that the ions form extended hydrogen bond networks.^58^ The key point is that although preferential association of ions has been seen in many studies of IL mixtures, long-lived discrete IPs have not been identified as the cause of these.

An attempt has been made to separate [C_2_C_1_im]_0.05_[C_8_C_1_im]_0.95_[Tf_2_N] and [C_2_C_1_im]_0.5_[C_8_C_1_im]_0.5_[Tf_2_N]_0.5_[EtOSO_3_]_0.5_ mixtures by fractional distillation under high vacuum.^59^ The vapour phase consisted of all possible combinations of neutral IPs from the liquid mixture, suggesting that there was no great preference of any particular cation for any particular anion. Interestingly, although it would be expected that the surface layer would be enriched with the [C_8_C_1_im]^+^ ion,^53,60^ the distillate from [C_2_C_1_im]_0.05_[C_8_C_1_im]_0.95_[Tf_2_N] was enhanced in the more volatile [C_2_C_1_im][Tf_2_N] constituents. Unfortunately, the [C_2_C_1_im]_0.5_[C_8_C_1_im]_0.5_[Tf_2_N]_0.5_[EtOSO_3_]_0.5_ decomposed.

One interesting solute is Mosher's salt (α-methoxy-α-trifluoromethylphenylacetic), a widely used chiral recognition agent.^61^ When dissolved in ILs with chiral cations, its ^19^F NMR spectrum clearly shows the formation of diastereomeric IPs, which must be sufficiently long-lived to be detected.^62^ This may be driven by strong hydrogen bonding of the carboxylate group of the Mosher's salt with the cations.

## Metal salts in ionic liquids

So far, I have only considered mixtures with large molecular ions, but often salts with small metal cations are dissolved in ILs. It has long been recognised that metals can form coordination complexes with IL anions,^63^ or react with imidazolium ions to form metal carbene complexes,^64^ but these are not the subject of this discussion.

Given the importance of lithium and sodium in battery technology, and the non-flammable nature of ILs, this has been a highly active area of research.^65^ The lithium cation acts as a hard Lewis acid in these systems which can interact with hard Lewis bases, as does the sodium ion to a lesser extent. These include the oxygen atoms of anions such as [NTf_2_]^−^, which has been noted to lead to the formation of [Li(NTf_2_)_2_]^−^ ions.^66^ Subsequent work on other metals and other IL anions has shown that the situation is complex and that the number of ions coordinating the metal centre depend on the charge and size of the metal ion as well as the IL anion.^67^ In a MD study of IL solutions of Na[FSA] (where [FSA]^−^ = bis(fluorosulfonyl)amide) in either [C_2_C_1_im][FSA] or [C_3_C_1_pyrr][FSA], {Na^+^–[FSA]^−^} IPs were identified, but so were larger clusters, such as [Na(FSA)_4_]^3−^ and [Na(FSA)_5_]^4−^.^68^

When the metal ion is introduced to the IL by dissolving its salt with a different anion to the IL, the cation has a choice of which anion it will interact with and any preference for one over the other could lead to the formation of IPs. Solutions of K[SCN] or [C_4_C_1_im][SCN] in [C_4_C_1_C_1_im][NTf_2_] were investigated using IR spectroscopy of the [SCN]^−^.^69^ The conventional FTIR spectra showed almost no difference in the spectra of the two solutes, which would usually be interpreted as there being the same solvation environment for the [SCN]^−^ in both the solutions. However, 2D-IR showed distinct evidence for K^+^–[SCN]^−^ interactions lasting for well over 100 ps, which is strong evidence for ion pairing. The solutions of the salts were only 30 mM, so the possibility of higher order clusters of solute ions was reasonably excluded.

A later study containing mixed cations and mixed anions used Raman and MD to investigate solutions of Li[NTf_2_] in mixtures of [C_3_C_1_pyrr][NTf_2_] and [C_2_C_1_im][DCA] (1 : 9 v/v).^70^ This study showed that the Li^+^ cations had a preference to be solvated by the [DCA]^−^ over the [NTf_2_]^−^, but in the form of [Li(NTf_2_)_*m*_(DCA)_*n*_]^(*m*+*n*−1)–^ (where *m* = 0–1 and *n* = 3–4) clusters, rather than neutral IPs.

Although these studies appear to contradict each other, this is not necessarily the case because the relative concentrations of the metal cation and the probe anions are so different. Both indicate a preference of the Li^+^ cation for the smaller and more basic anion in the system, with long-lived associations occurring as a result. It is possible that the K^+^–[SCN]^−^ is part of a larger cluster (*e.g.*, [K(SCN)(NTf_2_)]^−^) in the former study, or that IPs would form if the concentrations of Li^+^ and [DCA]^−^ were the same in the latter one. Further studies would be required to unravel this.

## Solutes with multiply charged ions

One way of encouraging ion pairing could be to use solute ions with greater than unity charge. Michael Ryan and Abderrahman Atifi have studied tetracyanoquinodimethane (TCNQ) in the ILs [C_4_C_1_im][BF_4_], [C_4_C_1_im][PF_6_] and [C_4_C_1_im][NTf_2_].^71^ This solute can hold charges from neutral to 2−. Using a combination of electrochemistry and spectroscopy and comparing to spectra they obtained in acetonitrile, they found no evidence for IPs for the [TCNQ]^−^ in any of the ILs, nor for [TCNQ]^2−^ in [C_4_C_1_im][BF_4_] or [C_4_C_1_im][PF_6_] solutions, but did in [C_4_C_1_im][NTf_2_]. In a study of the radical anion and dianion of dinitrobenzene (DNB) they identified similar strong interactions between [DNB]^2−^ and [C_4_C_1_im][PF_6_].^72^ However, it should be noted that while these studies clearly demonstrate stronger interactions between the cations and the dianions than the monoanions, it does not differentiate a particular cation that is interacting with the anions differently to the others.

Ruth Lynden-Bell built on her previous work to investigate doubly charged ions in [C_1_C_1_im]Cl to explore the possibility that a doubly charged anion in an IL composed of singly charged ions would be similar to that of singly charged ions in a neutral molecular solvent.^73^ What emerged was more complex, in that the likelihood of a 2+/2− IP forming was highly dependent on the size of the ions, with smaller ions forming IPs but IPs of larger ions being energetically disfavoured in the IL.

## Organic reactions in ionic liquids

One of the most active areas of research for ILs is their use as solvents for organic reactions and there have been many studies on how their structures can affect these.^74^ In fact, our original paper was inspired by a previous study in which we investigated the reaction of two salts, [electrophile][NTf_2_] and [C_4_C_1_im]Cl, in several ILs.^75^ In molecular solvents, this reaction proceeds by first forming the reactive IP {[electrophile]^+^–Cl^−^}, which goes on to complete the reaction, leading to complex reaction kinetics. In the ILs, simple pseudo-first-order kinetics were always seen, suggesting the absence of IPs.

Jin-Pei Cheng and coworkers have measured the absolute p*K*_a_ values of benzoic acids,^76^ sulfonamides^77^ and triphenylphosphonium and *N*-substituted pyridinium salts with anions ranging in size and basicity from Cl^−^ to tetrakis[3,5-bis(trifluoromethyl)phenyl]borate ([BAr^F^_4_]^−^).^78^ In [C_4_C_1_im][NTf_2_], [C_4_C_1_C_1_im][NTf_2_], [C_4_C_1_pyrr][NTf_2_] and [C_4_C_1_im][OTf] they found that the p*K*_a_s were entirely independent of the counterion of the dissolved salt. They concluded that the ILs were highly dissociating solvents, despite their low dielectric constants,^79^ and that the acids were present as “free ions” rather than IPs. However, it should be pointed out that the p*K*_a_s do change with changing the IL anion, so some degree of ion association is occurring between the solute acid and IL anion, but this is unlikely to be a 1 : 1 IP.

The reaction of CsF with alkyl mesylates to give the corresponding fluoride has been studied in mesylate ILs.^80^ By analogy with the reaction in polar solvents the authors stated that the reacting species was the {Cs^+^F^−^} IP. However, they also said that the reaction was accelerated by the IL anion being able to interact strongly with the Cs^+^ ion, so “freeing” the F^−^ ion, which equates to the breakup of any {Cs^+^F^−^} IP. In a subsequent paper,^81^ they support their claim using DFT calculations that show the IL cation [C_4_C_1_im]^+^, anion [OMs]^−^, F^−^, Cs^+^ and the leaving group of the substrate forming a compact cyclic structure. However, without calculations on a cluster containing more IL ions (see above), it is impossible to tell if this is what happens in the real solutions.

The photoinduced electron transfer (PET) reaction between pyrene and *N*,*N*-dimethylaniline (DMA) in a variety of ILs, shows an interesting combination of effects.^82^ This reaction proceeds by the formation of a geminate IP, which can either quench by back electron transfer or dissociate into separate radical ions that can be observed spectroscopically. In the more viscous ILs ([C_4_C_1_im][NTf_2_], [C_4_C_1_im][BF_4_] or [C_4_C_1_im][PF_6_]), no evidence of dissociation was found, which was interpreted as the quenching was sufficiently fast that the geminate IP never leaves the solvation cage formed by the ILs. In the less viscous [C_2_C_1_im][NTf_2_], a few radical ions do manage to separate whereupon they are longer lived than in molecular solvents. In order to exclude the possibility that this is just a viscosity effect, a more recent PET study of perylene with either DMA or dicyanoethylene compared the reactions in [C_2_C_1_im][DCA] (17 cP) with a DMSO/glycerol mixture of the same viscosity.^83^ The IL favoured the formation of free ions, while the DMSO/glycerol mixture favoured the recombination of the IP, confirming that this could be attributed to the strong screening of the geminate IP by the IL.

The electrochemical reduction of corannulene in [C_4_C_1_pyrr][NTf_2_] has been compared to the same process in acetonitrile.^84^ The authors determined that the mechanism followed a so-called ‘square scheme’ and, by analogy with a similar reaction of C_60_ in a mixture of benzonitrile with either [C_4_C_1_im][PF_6_], [C_4_C_1_im][BF_4_] or [N_3,2,1,1_][NTf_2_] (where [N_3,2,1,1_]^+^ = ethyldimethylpropylammonium),^85^ concluded that the reduction was controlled by ion pairing. However, the benzonitrile molecular solvent in the latter study would encourage ion pairing and the authors of that paper attributed their observations to “interaction of the fullerides with RTIL nanodomains, not merely by classical ion pairing”.

Asymmetric ion pair catalysis is a powerful tool to generate chiral molecules.^86^ Chiral ionic liquids have also received a great deal of attention for their potential for solvent induced enantioselective synthesis.^87^ However, where there is no added molecular co-solvent the enantioselectivity has been poor, suggesting that long-lived stereochemically active IPs are not formed in these solutions.

There are many examples of the effects of ILs on reactions being due to the IL ions interacting with charged species, whether these be starting materials, products, transition states or catalysts,^88^ and even of preferential interactions in mixtures of ILs,^89^ but these have rarely been attributed to the formation of discrete IPs.

## Bringing it all together

The examples that I have given above are non-exhaustive but do represent the range of studies of ion pairing in ILs that can be found in the literature. For neat ILs, the evidence for the existence of IPs is very weak, alternative explanations exist for the observations, or definitions that stretch the analogy with ion pairing in molecular solutions are required. Most studies point to the lack of ion pairing. The evidence for ion pairing is stronger for solutions of salts in ILs, for example when there is a strong driving force such as a size mismatch with the IL ions, but even these examples are rare. Ion pairing in ILs is certainly not commonplace. In spite of this, the term is commonly used in the literature and that is unlikely to change. However, I suggest to avoid confusion the terms IP and ion pairing should only be used when the evidence for these is strong.

The IP concept is one that was developed to explain the behaviours of dilute solutions of salts in molecular solvents.^14^ The question that I suggest we should be asking is not whether we can convince ourselves or not that IPs could be present in a particular IL or solution in these, but rather whether the transfer of this concept from molecular solvents to ILs provides any helpful insight into the multiple and multifaceted ion–ion interactions that occur in ILs.

## Author contributions

Tom Welton 100%.

## Conflicts of interest

There are no conflicts of interest to declare.

## Data Availability

There is no additional data associated with this article.
